# Type 1 diabetes: what is the role of autoimmunity in **β** cell death?

**DOI:** 10.1172/JCI164460

**Published:** 2022-10-17

**Authors:** Marc Y. Donath

**Affiliations:** Clinic of Endocrinology, Diabetes and Metabolism, University Hospital Basel, Basel, and Department of Biomedicine, University of Basel, Basel, Switzerland.

## Abstract

The current dogma of type 1 diabetes pathogenesis asserts that an autoimmune attack leads to the destruction of pancreatic β cells, with subsequent hyperglycemia. This dogma is based on islet autoantibodies emerging prior to the onset of type 1 diabetes. In this issue of the *JCI*, Warncke et al. report on their investigation of the development of hyperglycemia below the diabetes threshold as an early proxy of β cell demise. Surprisingly, they found that an elevation in blood glucose preceded the appearance of autoimmunity. This observation calls into question the importance of autoimmunity as the primary cause of β cell destruction and has implications for prevention and treatment in diabetes.

## Current views on the pathogenesis of type 1 diabetes

The current state of knowledge on the pathogenesis of type 1 diabetes proposes that insulin-producing β cells become altered and destroyed as a result of an autoimmune-mediated attack. This process is thought to occur in genetically predisposed individuals before the onset of diabetes, i.e., before metabolic decompensation, as evidenced by the appearance of autoantibodies directed against β cell antigens several months to years earlier (1). As a result, many therapeutic strategies to prevent and treat diabetes focus on modulating the immune system through antigenic vaccination or immunomodulatory treatments. Surprisingly, the vast majority of studies that have followed these strategies have shown a small initial benefit or no benefit at all (2–9). In cases where clinical benefit was observed, after an initial improvement in insulin production, the decline matched that observed in untreated patients with some delay.

Another puzzling observation concerns the histology of the pancreas of patients with type 1 diabetes (10). Indeed, fewer than 10% of islets become infiltrated, which is achieved with merely 15 leucocytes per islet and with only twice the quantity found in healthy controls. This observation is in stark contrast with histopathological findings associated with organs affected by classical autoimmune diseases, characterized by a massive influx of immune cells. It is noteworthy that the NOD mouse, which is considered the best animal model for human type 1 diabetes, has massive leukocyte infiltration in the islets, reflecting the well-documented immune-mediated destruction of β cells in this model. This discrepancy calls into question the value of the NOD mouse as a model for human pathology. Given the above, the etiology of type 1 diabetes is a matter of debate (11, 12), although the vast majority of researchers remain convinced that autoimmunity is the main driver of type 1 diabetes.

## Hyperglycemia precedes the appearance of autoimmunity

In this issue of the *JCI*, Warncke et al. (13) report the results of a longitudinal study in 1,050 children with a high genetic risk of developing type 1 diabetes. The researchers monitored a remarkable number of children over a long period of time, assessing changes in fasting and postprandial glycemia in parallel with islet autoantibody development. In the context of the pathogenesis of type 1 diabetes, blood glucose levels began to rise when β cell destruction resulted in insufficient insulin production to regulate glucose uptake. Typically, the first signs of β cell failure are detectable postprandially when more insulin is needed, which followed by fasting hyperglycemia as the disease progresses. Based on data obtained from surgical resection of the pancreas, at least 50% of β cells probably need to be destroyed to cause hyperglycemia (14). Strikingly, Warncke et al. observed a sharp rise in postprandial blood glucose two months before the detection of autoantibodies to β cell antigens (13). This finding led the authors to propose a different paradigm in the pathogenesis of the disease, in which a β cell insult precedes the development of autoimmunity (13).

An additional interesting aspect of the study led by Anette-G. Ziegler (13) was an unexpected temporary decrease in blood sugar levels observed in early childhood up to the age of 1 to 1.5 years. This outcome was influenced by sex, body weight, and genetic predisposition. It remains to be determined whether this temporary drop in blood glucose reflects differences in functional β cell mass, insulin sensitivity, or both and whether these may influence predisposition to diabetes (13).

## TIme to revisit the pathogenesis of type 1 diabetes?

The study by Warncke et al. (13) raises several questions about the pathogenesis of type 1 diabetes. First, what is the true role of autoimmunity, an important precipitating factor or a mere epiphenomenon? In the study, postprandial blood glucose began to rise two months before the onset of autoimmunity and continued to increase with the same rapidity after seroconversion, followed by an increase in fasting blood glucose. This dynamic does not argue for a major impact of autoimmunity on blood glucose changes and thus β cell demise. However, the study did not monitor patients to the onset of diabetes, and immune effector mechanisms may play a more important role later in the development of metabolic decompensation. Nevertheless, it is conceivable that the development of autoimmunity is the consequence of another as-yet-unidentified cause of β cell death. Indeed, massive cell death may trigger dendritic cells to become immunogenic antigen-presenting cells capable of activating the adaptive immune system (15). This phenomenon has been described, for example, after myocardial infarction (16). Of particular interest is the predisposition of patients with type 1 diabetes for activation of the adaptive immune response following myocardial infarction. This immune response may explain the excessive morbidity and mortality after heart infarction in patients with type 1 diabetes (16). Extrapolating these mechanisms to islets, a strong β cell insult, similar to ischemia during a myocardial infarction, followed by an exaggerated immune response due to a genetic predisposition may cause type 1 diabetes.

The association between insult and immune response raises a second question: if autoimmunity is a secondary event, what is the primary cause of β cell destruction in patients with type 1 diabetes? Multiple explanations may reflect the heterogeneity of type 1 diabetes, as shown by the differences in clinical manifestations according to age of onset (younger individuals have a more rapid destruction of β cells), the association or not with other diseases (Hashimoto’s thyroiditis, adrenal insufficiency, vitiligo, and celiac disease) within the framework of a polyglandular syndrome, and different risk factors (genetic, body weight, environmental).

**Figure 1:**
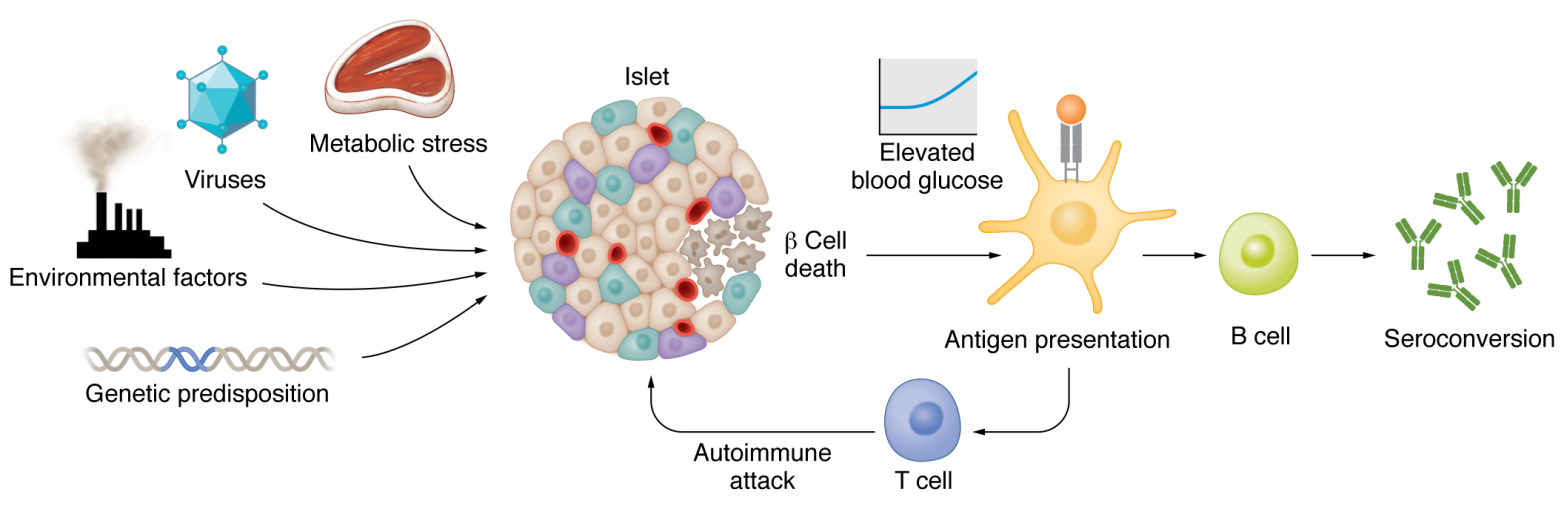
A model of factors that may lead to β cell destruction in type 1 diabetes. Various factors may drive β cell damage, including viral infection, metabolic stress, genetic predisposition, or environmental toxins. These insults could lead to β cell death and subsequent antigen presentation followed by autoimmunity to precipitate total β cell demise. The precise contribution of each factor and additional β cell toxic factors remains to be discovered.

A possible culprit for the initial cause of β cell death could be a virus. Indeed, infections with various viruses have been associated with the development of type 1 diabetes (17). A genetic or epigenetic predisposition leading to β cell degeneration is also conceivable. One can speculate that such an intrinsic predisposition could, for example, affect molecules involved in the cell secretory machinery, explaining the association of type 1 diabetes with other diseases involving secretory cells in the polyglandular syndrome. Factors related to obesity could also induce β cell death and thus also associate with type 2 diabetes. Indeed, metabolic stress leads to an activation of the innate immune system, with β cell death (18) and subsequent development of anti–islet-specific autoantibodies (19). Interestingly, the glucose genetic risk score used in the study conducted by Warncke et al. (13) is also associated with the risk of type 2 diabetes (20). Therefore, the etiology of β cell death in type 1 diabetes is likely multifactorial and may vary depending on the subtype (Figure 1). It may be mainly driven by autoimmunity in some cases, but in other cases, intrinsic predisposition to β cell degeneration, infection, environmental factors, and metabolic stress, alone or in combination, may be more important. It should be noted that Warncke et al. (13) studied a very specific population of children at high genetic risk for developing type 1 diabetes; other populations may differ in many ways with other subtypes.

Better characterization of the different subgroups of patients with type 1 diabetes and uncovering of the precise underlying etiology of β cell failure are essential for developing effective drugs to prevent or cure the disease. Currently, most efforts focus on modulating the immune system without distinguishing the possible underlying specific etiology and subgroup of type 1 diabetes. The findings in Warncke et al. (13) are a wake-up call, alerting us to precisely identify our enemy before waging an effective fight against it.

## Version 1 10/17/2022

Electronic publication
